# *KIF2C* affects sperm cell differentiation in patients with Klinefelter syndrome, as revealed by RNA‐Seq and scRNA‐Seq data

**DOI:** 10.1002/2211-5463.13446

**Published:** 2022-06-16

**Authors:** Haihong He, Tingting Huang, Fan Yu, Keyan Chen, Shixing Guo, Lijun Zhang, Xi Tang, Xinhua Yuan, Jiao Liu, Yiwen Zhou

**Affiliations:** ^1^ Clinical Laboratory Medicine Centre, Shenzhen Hospital Southern Medical University Shenzhen China

**Keywords:** hub genes, *KIF2C*, Klinefelter syndrome, single‐cell sequencing, sperm cell

## Abstract

Klinefelter syndrome (KS) is a leading contributor to male infertility and is characterised by complex and diverse clinical features; however, genetic changes in the KS transcriptome remain largely unknown. We therefore used transcriptomic and single‐cell RNA sequencing (scRNA‐seq) datasets from KS versus normal populations through the Gene Expression Omnibus (GEO) database to identify gene biomarkers associated with the occurrence of KS. We identified a total of 700 differentially expressed genes (DEGs) and completed Gene Ontology (GO), Kyoto Encyclopedia of Genes and Genomes (KEGG), enrichment pathway analysis and protein‐protein interaction (PPI) network analysis. A total of four unreported KS‐related hub genes (*KIF2C*, *MRPS2*, *RPS15* and *TSFM*) were identified. Validation of the single‐cell sequencing dataset showed that only *KIF2C* and *RPS15* were expressed in spermatocytes and that they were differentially expressed in sperm cells. Further construction of the developmental trajectories of these two genes in sperm cells showed that the *KIF2C* gene showed an upward trend throughout the differentiation and development of sperm cells. In conclusion, we report here that *KIF2C* may be closely related to the differentiation and development of sperm cells in KS patients, which is important for revealing the molecular mechanism of KS and conducting further studies.

## Graphical abstract

Klinefelter syndrome (KS) is a genetic cause of male infertility. We used DEGs, constructed PPI networks, screened for Hub genes and validated them with scRNA‐seq data to identify *KIF2C* as most associated with sperm development. This finding may lead to new options for the treatment of KS patients.

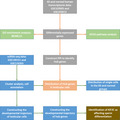

**Abbreviations**
BP: biological process
CC: cellular component
DEG: differentially expressed gene
DMNC: density of maximum‐neighbourhood component
DS: differentiating spermatogonia
EC: endothelial cell
EPS: early primary spermatocyte
ES: elongated spermatid
GEO: Gene Expression Omnibus
GO: gene ontology
HCL: human cell landscape
ICSI: intracytoplasmic sperm injection
KEGG: Kyoto encyclopedia of genes and genomes
*KIF2C*
: kinesin family member 2C
KS: Klinefelter syndrome
LC: leydig cell
LPS: late primary spermatocyte
MC: myoid cell
MCC: maximal clique centrality
MF: molecular function
MNC: maximum‐neighbourhood component
*MRPS2*
: mitochondrial ribosomal protein S2
NOA: non‐obstructive azoospermia
PPI: protein–protein interaction
*RPS15*
: ribosomal protein S15
RS: round spermatid
SC: sertoli cell
SCC: spermatogonial stem cell
scRNA‐seq: single‐cell RNA sequencing
TESE: testicular sperm extraction
*TSFM*
: Ts translation elongation factor, mitochondrial

Klinefelter syndrome, the most common type of sex chromosome abnormality in men, is recognised worldwide as the leading cause of male infertility [1]. Klinefelter syndrome has a prevalence of about 0.1–0.2% in newborn males, accounts for about 3% of male infertility patients and is responsible for about 10–12% of non‐obstructive azoospermia patients (NOA) [2]. This percentage is even higher in China: 16.5% in the south of the country, 14.1% in the north and 11.2% in Hong Kong. Data from our previous study in the region showed that KS accounted for 12.62% of NOA and was also the primary cause of male infertility [3].

The clinical phenotype of KS is complex and varied, typically characterised by sparse body hair, feminised gynecomastia, short penis and small, hard testicles [4]. About 80–90% of KS patients fall into the 47,XXY karyotype, with mosaicism and X chromosome structural abnormalities accounting for the remaining 10% [5]. From infancy to adulthood, and especially after puberty, the number of germ cells in KS patients is progressively reduced, with only a very small number or complete absence of germ cells in adulthood, eventually leading to male infertility [6]. With advances in assisted reproduction techniques such as testicular sperm extraction (TESE) and intracytoplasmic sperm injection (ICSI) combined, it is possible for 30–66% of KS patients to have biological offspring [7, 8, 9], but more than half of KS patients are not able to have offspring. Therefore, understanding the key factors that influence the process of sperm development is critical to help KS patients who are unable to have offspring.

The extra X chromosome in KS patients may be of paternal or maternal origin, with a slightly higher probability of being maternal than paternal (55% vs. 45%) [10, 11]. In patients with KS, current studies in the literature have found that additional genes on the X chromosome exhibit escape inactivation, allowing for abnormal gene expression in male individuals, resulting in the production of altered mRNA and gene products that may correlate with the severity of the KS phenotype. The pathogenesis of spermatogenesis disorders due to abnormal gene expression in KS patients is poorly understood and still needs further elucidation [12, 13].

In the current study, we utilised gene expression profiling microarray transcriptomic data and scRNA‐seq data from the GEO database. Differentially expressed genes were obtained by analysing gene expression transcriptomic data from KS and normal human testicular tissue. We then performed GO and KEGG enrichment analyses and further identified hub genes by PPI network analysis. We identified hub genes expressed in sperm cells by integrating scRNA‐seq data from KS and normal human testicular cells. The role of hub genes in sperm development was revealed by proposed temporal sequence analysis.

## Materials and methods

### Research data

GEO (http://www.ncbi.nlm.nih.gov/geo) is one of the free and data‐rich public genomic databases, containing various types of gene microarray data, microarray and single‐cell sequencing data, etc. The gene expression transcriptome data for this study were derived from the GSE103905 and GSE103613, with the GSE103905 data derived from three adult KS samples and four adult normal controls and the GSE103613 data derived from four fetal KS samples and five adult controls [14, 15]. The scRNA‐seq data were derived from the GSE130151 and GSE124263, with the GSE130151 dataset derived from testicular tissue from one adult KS and the GSE124263 dataset consisting mainly of four normal human testicular cells [16, 17].

### Screening for DEGs

Differentially expressed genes were identified by the limma package (version 3.46.0) of r software (version 4.02) with screening thresholds set at ¦log2 FC¦ > 1.5 and *P* < 0.05.

### GO and KEGG enrichment pathway analyses

Gene ontology and KEGG enrichment pathway analyses were performed on DEGs using the david (version 6.8) online website (https://david.ncifcrf.gov/). GO functional analyses were performed mainly by BP, MF and CC and using the ggplot2 package (version 3.3.5) for bubble map visualisation analysis.

### PPI network construction and identification of hub genes

The NetworkAnalyst website (https://www.networkanalyst.ca/) [18] was used to construct a testis tissue‐specific PPI network of DEGs. Subsequently, the data after NetworkAnalyst website analysis were imported into cytoscape software (version 3.6.1) to construct the PPI network. The four calculation methods degree, density of maximum neighbourhood component (DMNC), maximal clique centrality (MCC), and maximum‐neighbourhood component (MNC) in the cytohubba plug‐in were used to identify hub genes in the PPI network.

### Integration of single‐cell sequencing data, cell clustering and annotation, hub genes in testis clustered cells

ScRNA‐seq datasets were analysed using the seurat (version 4.0.2) and harmony package (version 1.0). Marker genes for cell clustering were obtained using the FindAllMarkers function, and the singler package (version 1.4.1) was automatically annotated in combination with manual querying of the Human Cell Landscape (HCL) database (http://bis.zju.edu.cn/HCL/) for cell clustering.

### Constructing trajectories of hub genes in single‐cell datasets

monocle3 (version 1.0.0) is an r package dedicated to the analysis of scRNA‐seq data, providing a variety of functions such as clustering, pseudotime, differential analysis and more. The developmental trajectory of sperm cells and hub genes is constructed by using functions such as reduce_dimension, learn_graph and plot_genes_in_pseudotime.

## Results

### Identification of DEGs in the transcriptome dataset

**Fig. 1:**
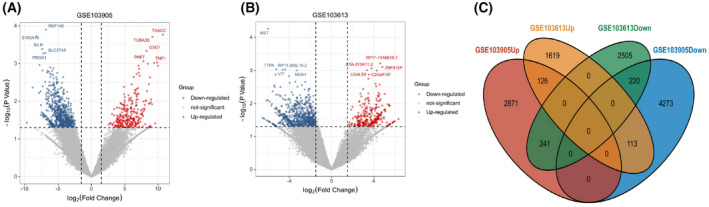
Identification of differentially expressed genes (DEGs) in Klinefelter syndrome and normal individuals, blue represents down‐regulated genes and red represents up‐regulated genes. (A) Volcano plot showing DEGs in the GSE103905 dataset. (B) Volcano plot showing DEGs in the GSE103613 dataset. (C) Venn plot showing DEGs in the two datasets overlapping. [Colour figure can be viewed at wileyonlinelibrary.com]

A total of 7844 DEGs were obtained in the GSE103905 dataset, containing 4606 down‐regulated genes and 3238 up‐regulated genes (Fig. 1A), while 4824 DEGs were obtained in the GSE103613 dataset, containing 2966 down‐regulated genes and 1858 up‐regulated genes (Fig. 1B). The Venn diagram showed 700 DEGs overlapping between the two datasets (Fig. 1C).

### GO and KEGG enrichment analysis

**Fig. 2:**
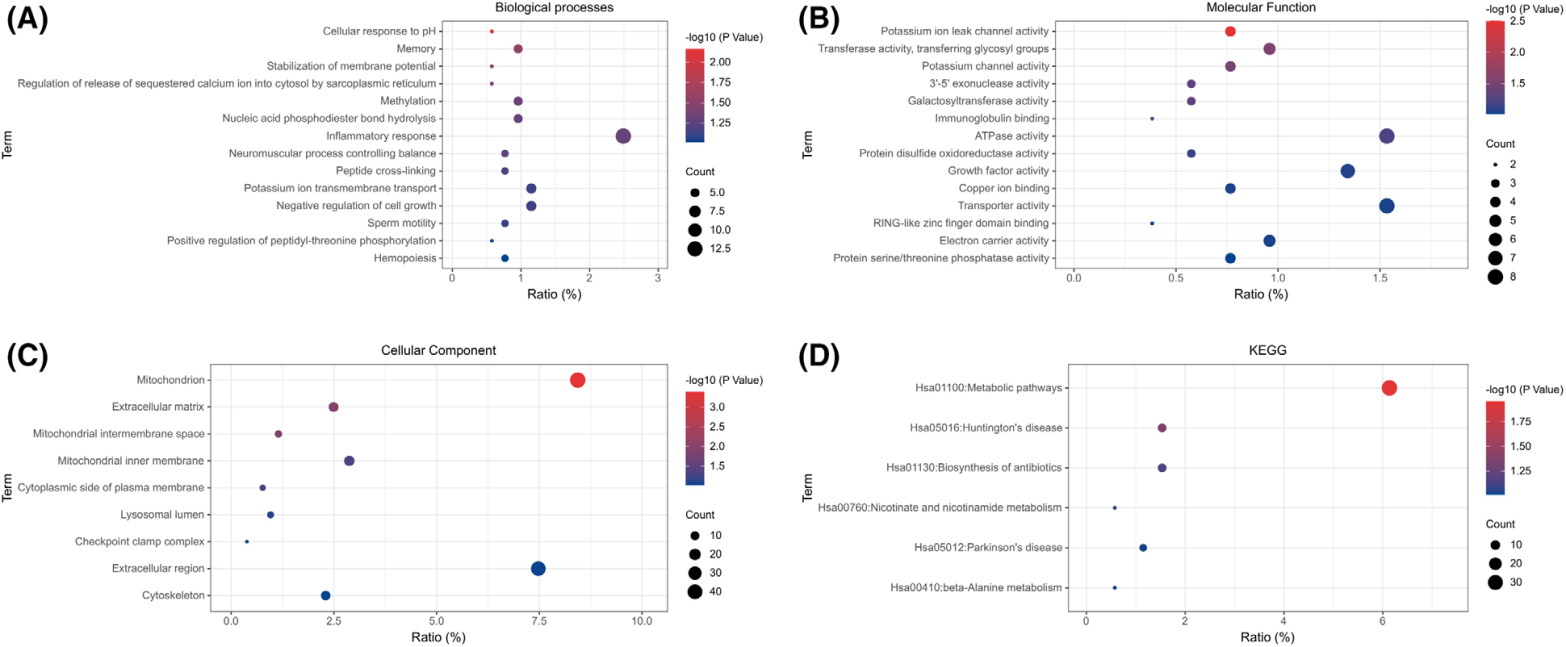
Gene ontology and Kyoto encyclopedia of genes and genomes (KEGG) enrichment analysis of differentially expressed genes. (A) BP enrichment analysis. (B) MF enrichment analysis. (C) CC enrichment analysis. (D) KEGG signalling pathway enrichment analysis. [Colour figure can be viewed at wileyonlinelibrary.com]

DEGs were analysed by david online analysis for GO functional enrichment and KEGG signaling pathway analysis. In the BP category, significant enrichment was found in stabilisation of membrane potential, memory and methylation (Fig. 2A). In the MF category, significant enrichment was found in potassium ion leak channel activity, ATPase activity (Fig. 2B). In the CC category, the mitochondrion, extracellular matrix and mitochondrial intermembrane space were significantly enriched (Fig. 2C). In the KEGG enrichment analysis, it was found that the signalling pathways that may affect spermatogenesis are as follows: metabolic pathways, Huntington's disease and nicotinamide metabolism (Fig. 2D).

### Construction of PPI networks and identification of hub genes

**Fig. 3:**
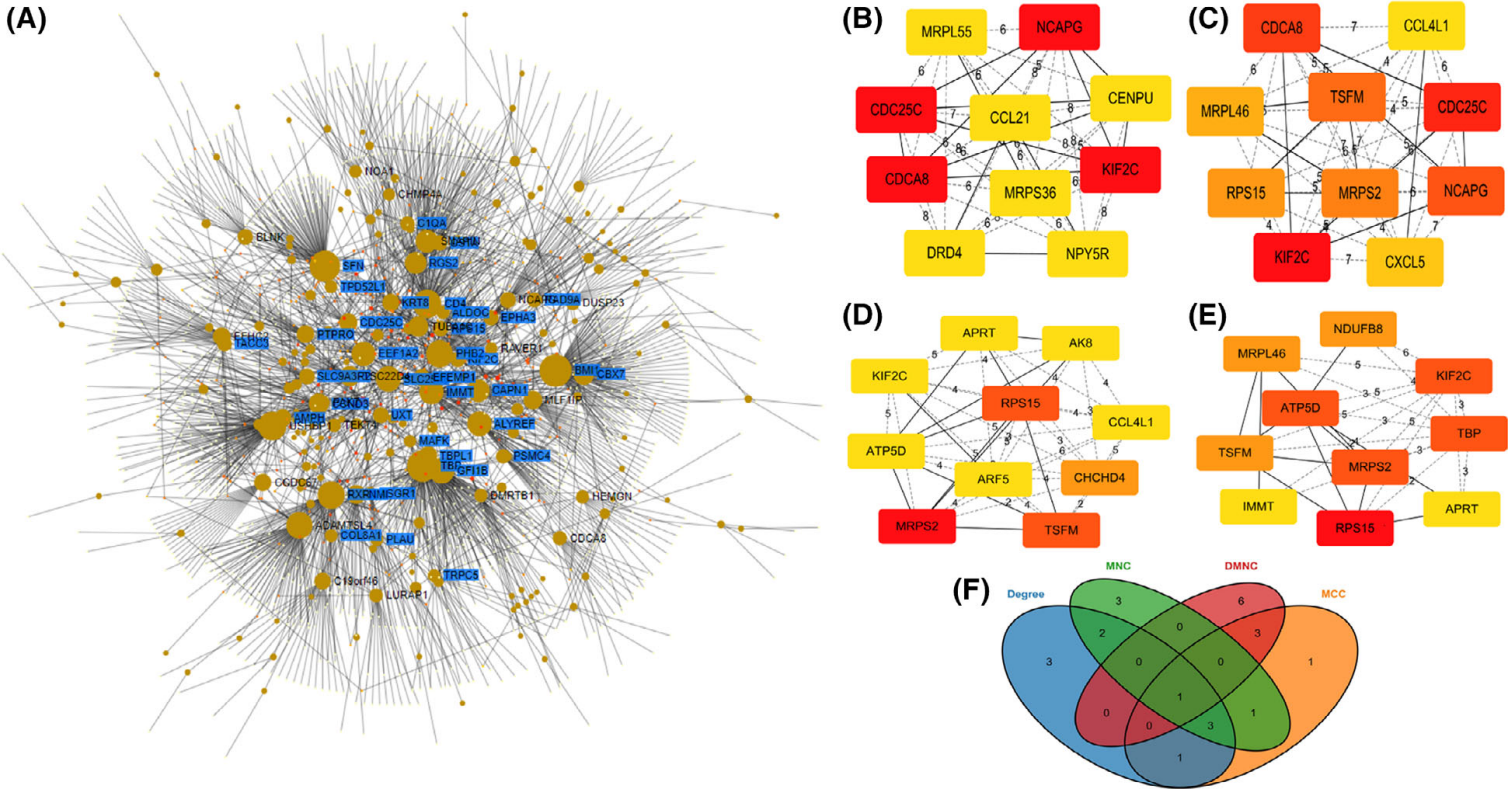
Testicular tissue‐specific PPI network and identification of hub genes. (A) Testicular tissue‐specific PPI network of differentially expressed genes. (B) TOP 10 key genes of the degree algorithm. (C) TOP 10 key genes of the MCC algorithm. (D) TOP 10 hub genes of the MNC algorithm. (E) TOP 10 key genes of the DMNC algorithm. (F) Venn diagram visualisation of four hub genes. [Colour figure can be viewed at wileyonlinelibrary.com]

The testicular tissue‐specific PPI networks of DEGs were analysed through the NetworkAnalyst website, and then, these networks were imported into cytoscape to form a PPI of 254 nodes and 340 edges (Fig. 3A). We used the Degree, DMNC, MCC and MNC algorithms in the cytohubba plugin to obtain the Top 10 key genes for each algorithm (Fig. 3B‐E). Finally, four genes (*KIF2C*, *MRPS2*, *RPS15* and *TSFM*) were repeated in more than three algorithms and they were considered as hub genes (Fig. 3F).

### Expression of hub genes in sperm cells

**Fig. 4:**
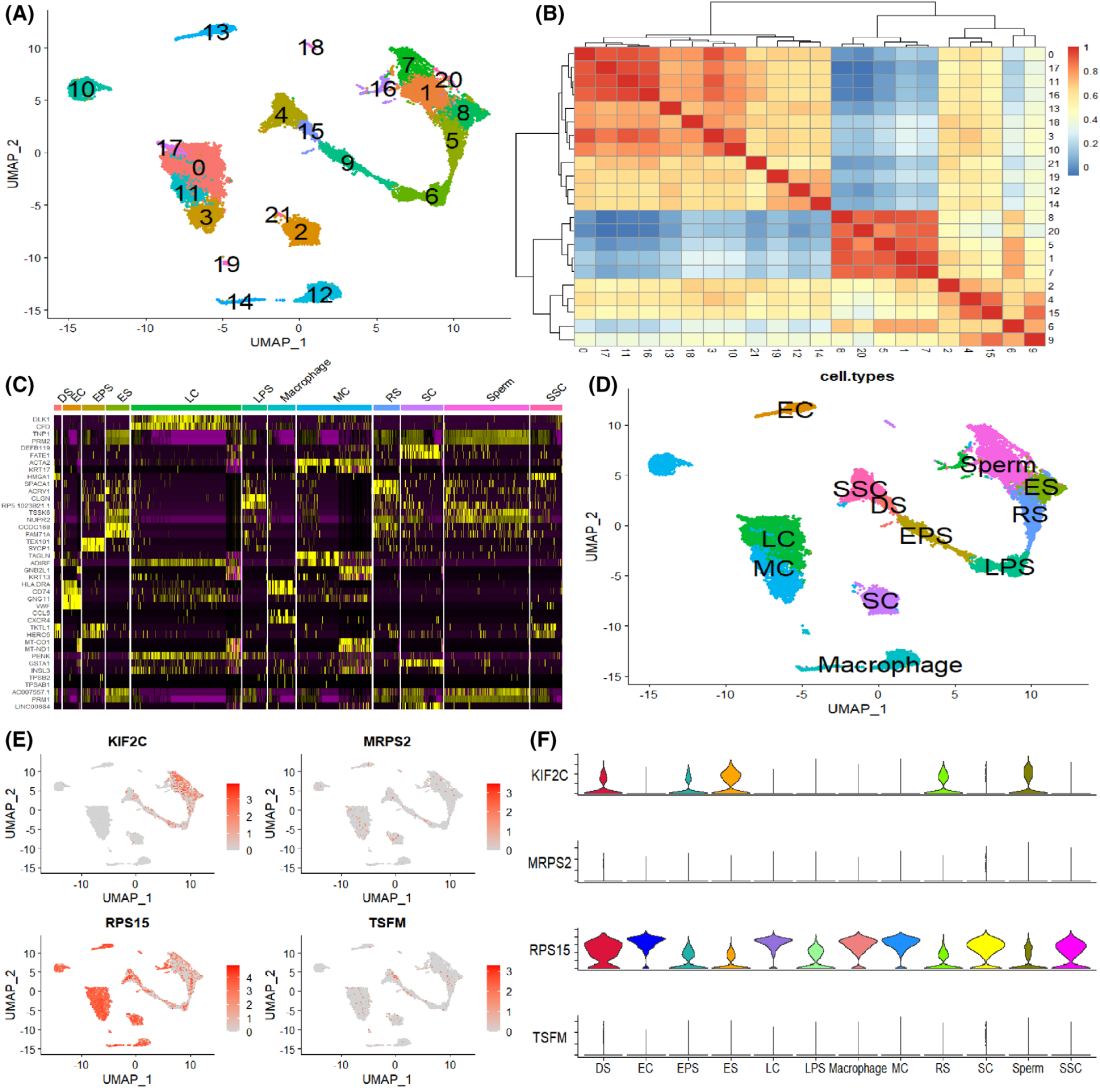
Expression of hub genes in testicular cells. (A) Integration of two single‐cell sequencing datasets and UMAP visualisation of 20 clustered cells. (B) Heatmap of correlation between cell clusters. (C) Heatmap presenting marker genes for cell clustering. (D) UMAP plots of 12 testicular cells. (E) Distribution map visualising the expression of four hub genes in testicular cells. (F) Violin plot showing the expression of hub genes in 12 testicular cells. [Colour figure can be viewed at wileyonlinelibrary.com]

To further investigate the expression of the four hub genes in sperm cells, we integrated scRNA‐seq data from testicular tissues of normal human and KS patients and constructed a single‐cell atlas formed by clustering 20 cells (Fig. 4A). A total of 12 sperm cells were identified by similarity heatmap of cell clustering and marker genes (Fig. 4B,C). They were as follows: leydig cell (LC), myoid cell (MC), macrophage, sertoli cell (SC), endothelial cell (EC), spermatogonial stem cell (SSC), early primary spermatocyte (EPS), differentiating spermatogonia (DS), late primary spermatocyte (LPS), round spermatid (RS), elongated spermatid (ES) and sperm (Fig. 4D). Only *KIF2C* and *RPS15* were expressed in the cell clusters, with *RPS15* being expressed in all cells, whereas *KIF2C* gene expression was cell specific and only expressed in the DS, ES, RS, EPS and sperm (Fig. 4E,F). We therefore suggest that *RPS15* and *KIF2C* have a more significant role in influencing sperm development.

### Differential expression of hub genes in KS and normal groups

**Fig. 5:**
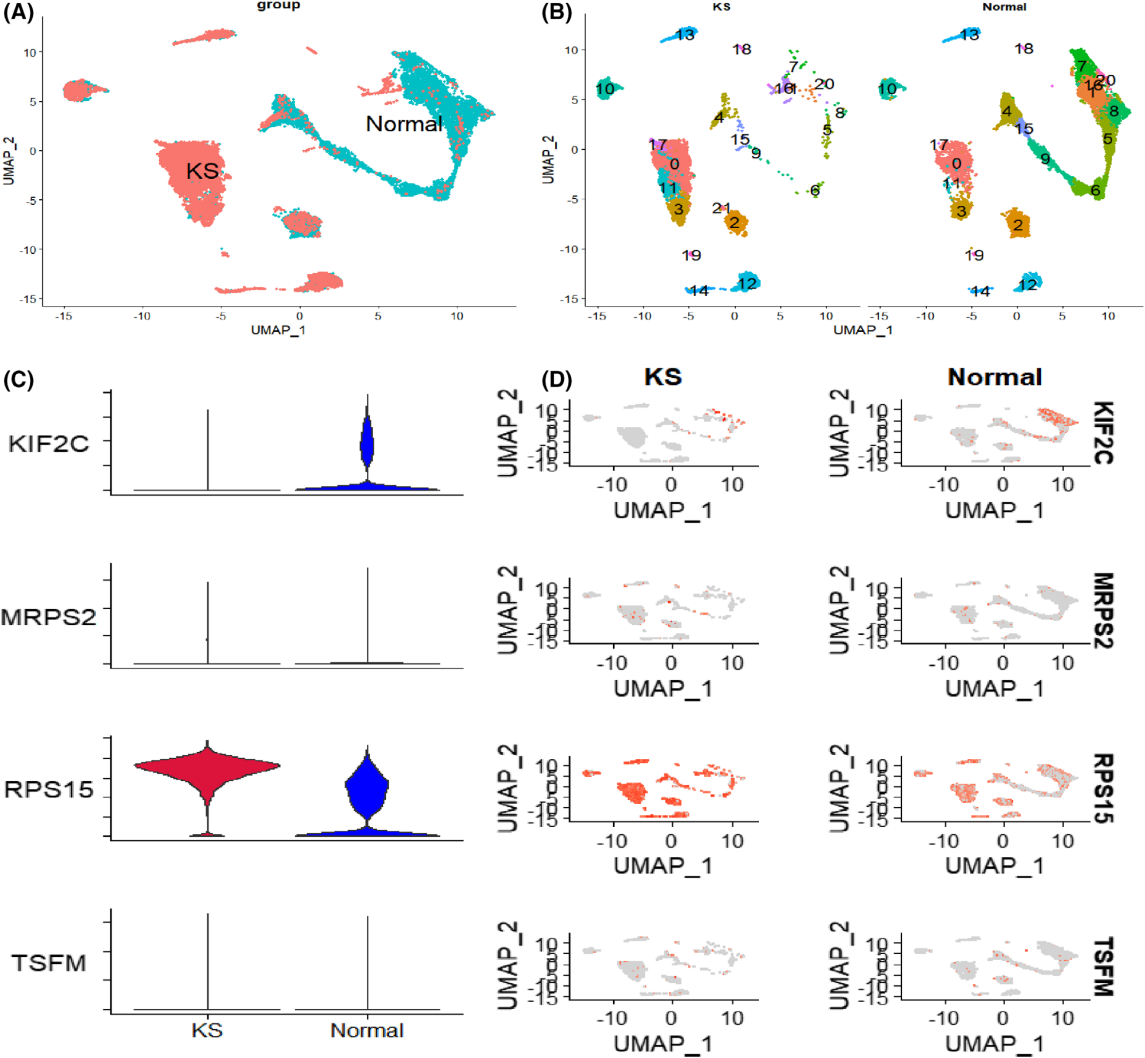
Expression of hub genes in the Klinefelter syndrome (KS) and normal groups. (A) Overall distribution of the KS and normal groups visualised by UMAP after integration of the two sets of single‐cell data. (B) Composition of cell clusters in KS and normal groups. (C) Violin plot showing the difference in expression of hub genes between the two groups. (D) Plot of the distribution of hub genes in the cell clusters in the two groups. [Colour figure can be viewed at wileyonlinelibrary.com]

In the single‐cell dataset, where sperm cells were rare in KS patients, testicular somatic cells (SC, MC, EC and LC) and immune cells were predominant, whereas normal human testicular cells contained 12 species of testicular cells covering the whole process of spermatogenesis (Fig. 5A,B). The analysis showed that the *RPS15* gene in KS was expressed at higher levels in all testicular cells than in normal subjects, whereas the expression level of the *KIF2C* gene was only expressed in the normal group and almost absent in the KS group (Fig. 5C,D).

### Pseudotime analysis of KIF2C and RPS15 in spermatocytes

**Fig. 6:**
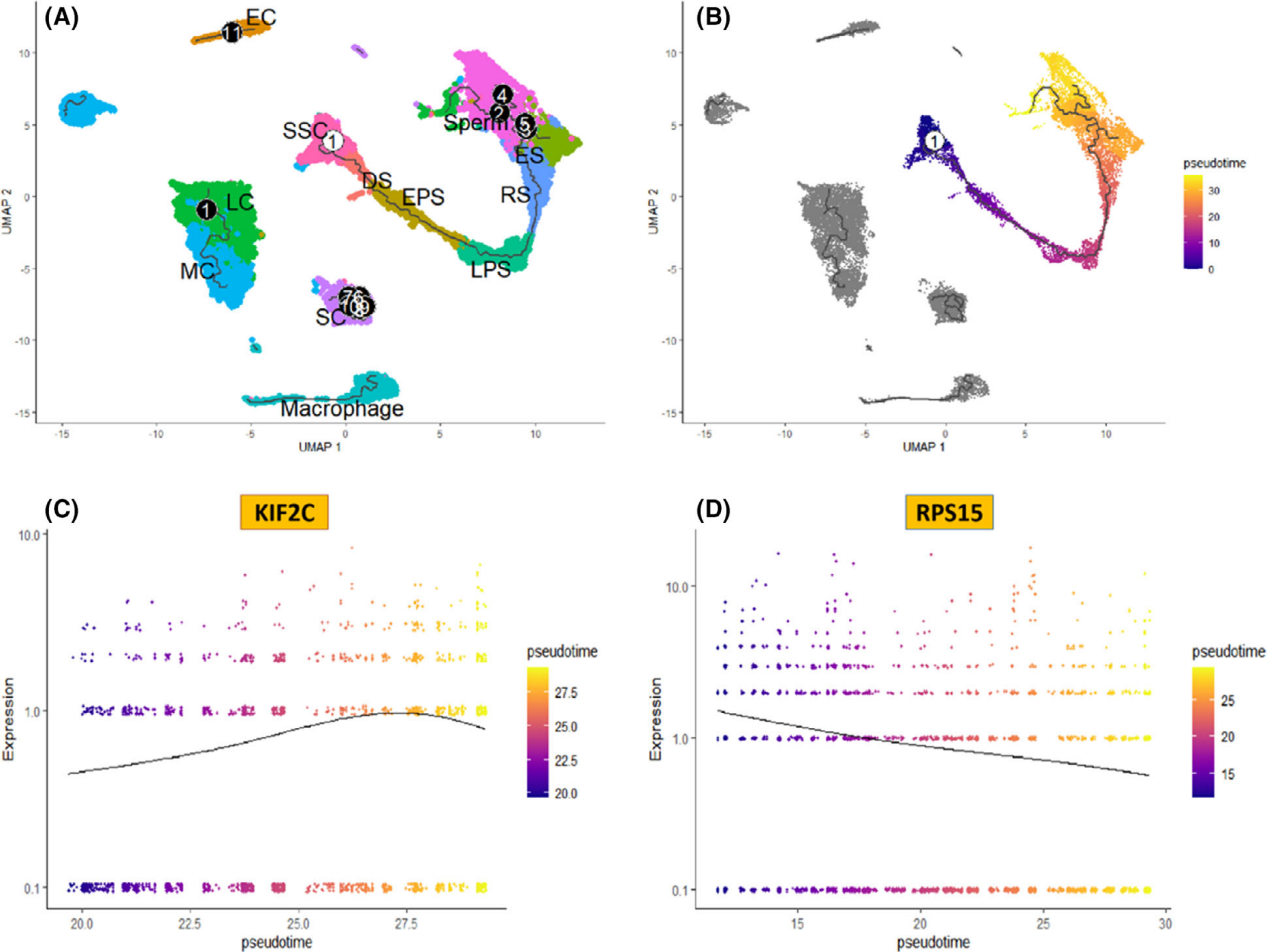
Pseudotime analysis of testicular cell development. (A) Developmental trajectory of testicular cells, numbers in white on a black background indicate the branch that occurs in the developmental trajectory of testicular cells. (B) Developmental trajectory of sperm cells, ① in black on a white background indicates that sperm development begins at the SSC. (C) Analysis of the trajectory of *KIF2C* throughout the development of sperm cells. (D) Analysis of the trajectory of *RPS15* in the full development of sperm cells. [Colour figure can be viewed at wileyonlinelibrary.com]

We verified that *KIF2C* and *RPS15* were differentially expressed in sperm cells from the KS and the normal group, and it was necessary to accurately capture the developmental trajectories of these two genes in sperm cells. The developmental trajectory of sperm cells was constructed using monocle3 software, and the results showed that a total of 11 branches were present in the sperm cells, and the branches were mainly distributed throughout the development from ES to sperm and in SC cells (Fig. 6A). During spermatogenesis, we successfully identified the initiating cell as SSC and the terminal cell as sperm (Fig. 6B). We constructed trajectories of *KIF2C* and *RPS15* in sperm development, and the results showed that *RPS15* showed a downward trend from SSC to sperm formation. The expression of *KIF2C* showed an upward trend from SSC to sperm formation, indicating that *KIF2C* plays a crucial role in sperm development (Fig. 6C,D).

## Discussion

Klinefelter syndrome is an important cause of non‐obstructive azoospermia (NOA) and oligospermia, which is accompanied by impaired spermatogenesis, with 90% of patients presenting with azoospermia and 10% with severe oligospermia [19, 20]. Furthermore, the effects of KS mosaicism may not be limited to germ cells；Ivan scholars [21] had found that KSM may be a factor in the pathogenic cascade in psychiatric and neurodegenerative disorders, particularly in cases where two or more X chromosomes are overrepresented. Moreover, mosiaicism is likely to be tissue specific [22], and therefore our focus in this study was primarily on single‐cell sequencing of testicular tissue. Not all X‐linked genes are completely inactive, with 12% escaping X‐chromosome inactivation and a further 15% having different X‐chromosome inactivation status in different individuals, tissues or cells. The expression of these genes from the second and other inactive X chromosomes may characterise many of the symptoms in Klinefelter males who have both inactive X and Y chromosomes [23]. However, the effect of the extra X chromosome on germ cells remains a mystery, and the process of KS leading to spermatogenesis disorders is a complex process involving multiple regulatory mechanisms, of which the study of gene regulation at the transcriptional level has become a hot topic in recent years and has helped to elucidate the molecular mechanisms underlying KS spermatogenesis disorders.

Several studies have shown that alterations in transcriptome genes affect the development of KS spermatocytes with different clinical phenotypes [24]. The transcriptome data from the current study showed that in the GSE103905 dataset, there were 7844 DEGs with 4606 down‐regulated genes and 3238 up‐regulated genes. In contrast, in the GSE103613 dataset, there were 4824 DEGs, 2966 down‐regulated genes and 1858 up‐regulated genes, with 700 DEGs overlapping between the two datasets. Subsequently, the GO functional annotated BP classification of DEGs resulted mainly in stabilisation of membrane potential, memory and methylation. Some of these studies have confirmed that KS is associated with predominantly genome‐wide hypermethylation and less hypomethylated genomic regions [25, 26].The MF classification results are mainly in potassium ion leak channel activity and ATPase activity. The CC classification results are mainly in mitochondrion, extracellular matrix and mitochondrial intermembrane space. KS patients with motor dysfunction and cognitive‐psychological impairment are more likely to suffer from depression, anxiety, schizophrenia, autism and attention deficit disorder, and 20–50% of patients have intentional tremor [27, 28].In addition to this, KS patients have a range of complications and are more likely to develop metabolic‐related diseases such as type 2 diabetes, dyslipidaemia, fatty liver, peripheral vascular disease, thromboembolic disease and endocrine‐related diseases [29]. These proven clinical signs and their complications are highly consistent with our results.

The testicular tissue‐specific PPI network of DEGs was constructed and visualised by NetworkAnalyst. Then, four algorithms, Degree, DMNC, MCC and MNC, were used to obtain the top 10 genes for each algorithm using the cytohubba plugin in cytoscape software, for a total of 40 genes, and four genes, *KIF2C*, *MRPS2*, *RPS15* and *TSFM*, were screened for the first time as hub genes, which may be closely related to spermatogenic disorders in KS patients.

To investigate in depth the expression of these four hub genes in testicular cells, we obtained 20 clustered cells by integrating testicular cells from GSE130151 and GSE124263 using the seurat package of r software. Subsequently, a total of 12 spermatocytes were identified by cell annotation using information from the singler and HPL databases [30]. Our results showed that only *KIF2C* and *RPS15* of the four Hub genes were expressed in cell clusters, with *RPS15* being expressed in all cell clusters, while the *KIF2C* gene had cell‐specific expression and was only expressed in spermatocytes, where cell specificity existed. Further differentiation by different populations showed that the *RPS15* gene in KS was expressed at higher levels in all testicular cells than in normal individuals, whereas the *KIF2C* gene was expressed at levels only in the normal group and almost absent in the KS group. The above studies suggest that the expression of these two hub genes differs in the process of sperm development.

To investigate in depth the role of *KIF2C* and *RPS15* in sperm development, we successfully constructed trajectories of *RPS15* and *KIF2C* genes in sperm development using the monocle3 package. *RPS15* showed a downward trend from SSC cells to sperm. The *RPS15* gene encodes a protein belonging to the S19P family of ribosomal proteins, which is a component of the 40S subunit [31]. As is typical for genes encoding ribosomal proteins, there are multiple processed pseudogenes of this gene dispersed through the genome. Due to the decreasing trend of *RPS15* during germ cell development, the *RPS15* gene has mostly been found to be activated in tumours, such as leukaemia, chronic lymphocytic leukaemia and prostate cancer, but no studies have been seen to correlate it with sperm development. Therefore, we believe that further corroboration is needed to determine whether it affects sperm cell development [32, 33]. The *KIF2C* encodes a kinesin‐like protein that functions as a microtubule‐dependent molecular motor. The encoded protein depolymerises microtubules at the positive end, thereby facilitating mitotic chromosome segregation [34]. Our study found that the expression level of the *KIF2C* gene was significantly lower in KS testicular tissue than in normal subjects, and that *KIF2C* showed a progressive increase during sperm development, indicating that the gene plays an important role in the differentiation and development of germ cells. It has been shown in animal experiments that *KIF2C* plays an important role in mitosis and meiosis in sperm cells [35]，and that mutations in *KIF2C* are closely associated with sperm cell differentiation and development, and may even result in aneuploid gametes [36]. Whereas cytogenetic alterations in KS are mainly dominated by abnormal sex chromosome numbers (aneuploidy variants), we suggest that *KIF2C* may be the key gene affecting sperm differentiation and development in KS. Therefore, the development of drugs related to the *KIF2C* gene and its encoded protein may provide a new direction and idea for the treatment of patients with KS azoospermia or oligospermia.

## Conclusion

In conclusion, in this study, 700 DEGs and their GO and KEGG enrichment pathways were identified in KS and normal populations, and four hub genes were screened out, and finally, the *KIF2C* gene was identified as possibly playing an important role in sperm development by constructing developmental trajectories of sperm cells. However, the mechanism by which it affects sperm development has not been clarified and warrants further in‐depth study. The new findings of this study provide important insights into the infertility of KS, and *KIF2C* may be a biomarker leading to impaired sperm development in KS, providing a strong basis for accurate diagnosis and precise treatment of KS.

## Conflict of interest

The authors declare no conflict of interest.

## Author contributions

HH, TH, YZ and FY conceived and designed the project; KC, SG and LZ acquired the data; XT and JL analysed and interpreted the data and HH and XY wrote the study.

## Data Availability

The data are available by contact with the corresponding authors.
